# Fatal pneumonia in a patient with Kearns-Sayre syndrome case report and literature review

**DOI:** 10.3389/fmed.2025.1575384

**Published:** 2025-07-31

**Authors:** Jiaqi Zhang, Ziqin Song, Huimin Zhou, Yaru Zhou, Shuchang Wang

**Affiliations:** Department of Endocrinology, Third Hospital of Hebei Medical University, Shijiazhuang, China

**Keywords:** Kearns-Sayre syndrome (KSS), COVID-19, ptosis, hypopituitarism, atrioventricular block, type 1 diabetes, mitochondrial DNA

## Abstract

Kearns-Sayre syndrome is a mitochondrial DNA deletion disorder, classically characterized by a triad of onset before the age of 20, pigmentary retinopathy, and chronic progressive external ophthalmoplegia (CPEO). The condition is frequently associated with third-degree atrioventricular block, endocrine dysfunction, and short stature. Here, we report a case of KSS presenting with a progressive clinical course that began at birth with ptosis and later developed type 1 diabetes, hypopituitarism, and third-degree atrioventricular block. Genetic analysis of a urine sample revealed a large-scale mitochondrial DNA deletion, confirming the diagnosis of KSS. The patient ultimately died from severe pneumonia precipitated by COVID-19 infection.

## 1 Introduction

Kearns-Sayre syndrome is a rare disorder caused by large-scale mitochondrial DNA deletions and classified as a form of mitochondrial myopathy (1). It occurs predominantly in sporadic cases (2), with the first case dating back to 1958 (3). The prevalence of mitochondrial diseases, including KSS, shows marked regional variation, largely influenced by genetic background and diagnostic accessibility. Population-based studies have reported rates of 23 per 100,000 in Northeast England, 1.6 in Finland, and 2.9 in Japan, with localized European peaks reaching up to 8.4 per 100,000 (4–7). Comparable or intermediate rates have been observed in other European and Oceanian populations (8). A territory-wide survey in Hong Kong estimated mitochondrial disease prevalence at 1.02 per 100,000, rising to 1.6 per 100,000 when mild or asymptomatic cases were included (9). Furthermore, a multicenter study in mainland China (*n* = 1351) reported that KSS accounted for 2.8% of mitochondrial disease diagnoses (10). These findings align with global prevalence estimates for KSS, which range from 1 to 3 per 100,000 individuals (11). However, comprehensive population-based data for mainland China remain unavailable.

Clinically, KSS typically presents before the age of 20 and is characterized by the classic triad of CPEO, bilateral ptosis, and pigmentary retinopathy, often accompanied by cardiac conduction abnormalities (3, 12, 13). Additional clinical features frequently include muscle weakness, cognitive impairment, and various endocrine dysfunctions (1, 12). Pathologically, large-scale mitochondrial DNA deletions are detectable in multiple tissues, with mosaicism between wild-type and mutant mitochondrial DNA within cells, contributing to the wide spectrum of phenotypic variability seen in KSS patients (14). The types of mitochondrial DNA deletions in KSS patients are highly heterogeneous. Most cases are caused by a single large-scale deletion ranging from approximately 1.1 to 10 kb, with the 4.9 kb “common deletion” (m.8483_13459del) being the most frequently reported (15). Studies have demonstrated that the size and gene content of the deletion correlate with phenotypic severity: larger deletions involving a broader set of oxidative phosphorylation complex subunits and mitochondrial tRNAs are more likely to cause early onset and multisystem involvement, increasing the likelihood of a full KSS phenotype, whereas smaller deletions or lower mutant loads are more often associated with milder presentations, such as isolated CPEO (16). Although deletion breakpoints vary, they tend to cluster within specific hotspot regions–one recent cohort study found that 96% of patients had deletions involving the MT-ND5 gene (11). Furthermore, the heteroplasmy level significantly influences disease risk, with higher proportions of deleted mitochondrial DNA in blood being associated with earlier disease onset (11).

In this report, we present a case of KSS in a patient exhibiting clinical features of ptosis, type 1 diabetes, hypopituitarism, and third-degree atrioventricular block. Genetic testing confirmed the presence of a large mitochondrial DNA deletion. The patient ultimately succumbed to a COVID-19 infection–an uncommon but serious outcome in individuals with KSS. This case highlights the need for increased clinical vigilance regarding the potential complications in KSS patients, particularly in the setting of systemic viral infections such as COVID-19.

## 2 Case description

### 2.1 Case overview

**FIGURE 1:**
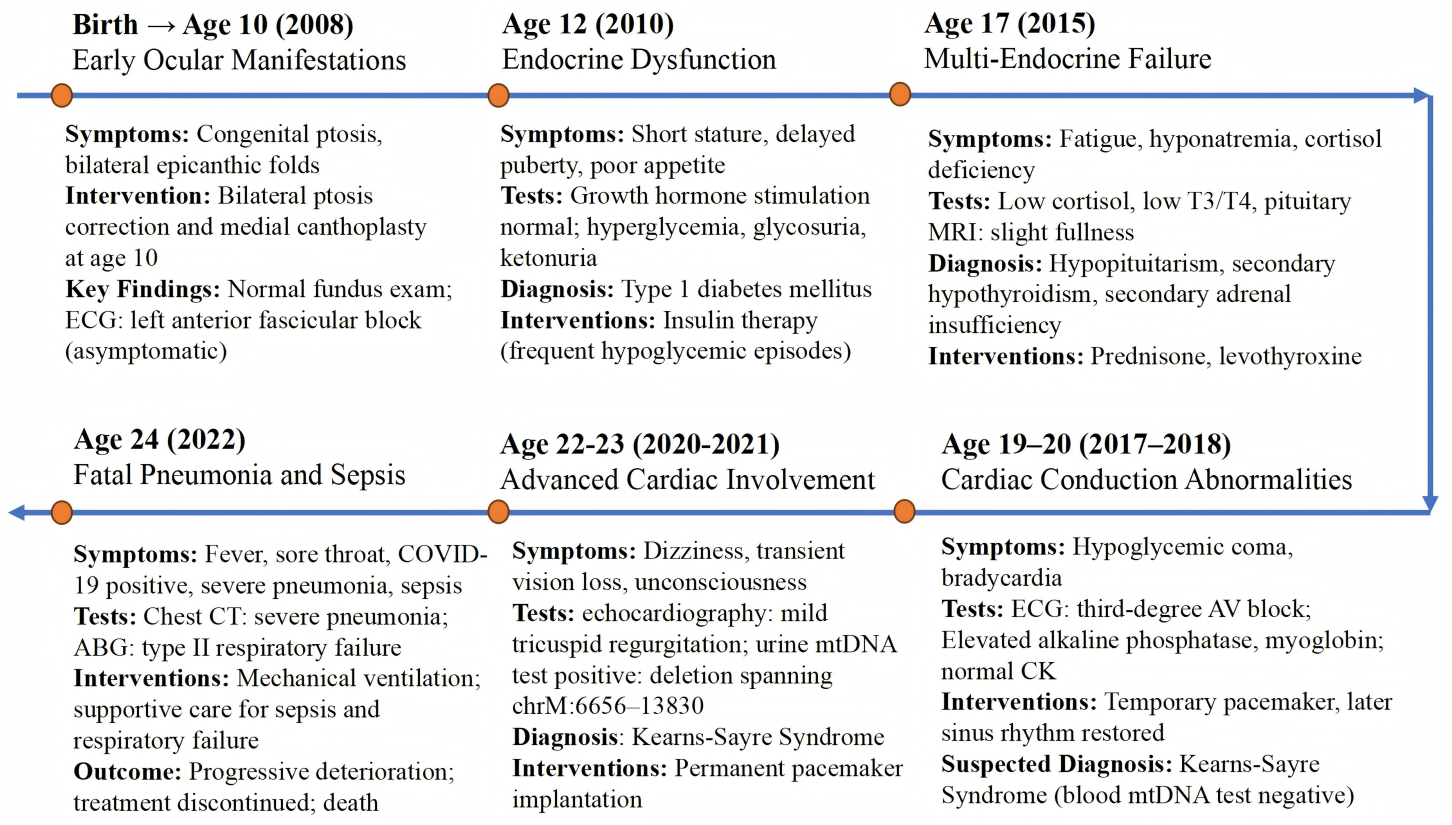
Timeline of the patient’s clinical course. Timeline illustrating the patient’s key clinical manifestations, diagnostic tests, therapeutic interventions, and disease progression leading to the final diagnosis of Kearns–Sayre syndrome and fatal outcome.

**FIGURE 2:**
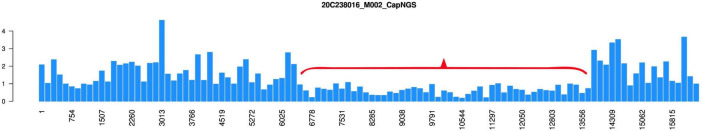
Genetic test results for the urine sample of the patient. The X-axis denotes nucleotide positions across the mitochondrial genome (chrM: 1–16569), while the Y-axis represents the normalized copy ratio (target gene coverage/reference gene coverage). The red bracket highlights a region of significant copy number reduction (chrM: 6656–13830), indicating a potential large-scale mitochondrial DNA deletion.

The patient described in this case report was born at term with a birth weight of 2,500 g and congenital bilateral ptosis with epicanthic folds; fundoscopic examination was unremarkable, and an asymptomatic left anterior fascicular block was identified in childhood (Figure 1). At age 10, he underwent bilateral ptosis correction and medial canthoplasty. By age 12, he presented with short stature (146.2 cm, −2.1 SD), poor appetite, delayed puberty, and hyperglycemia (fasting 5.96 mmol/L; postprandial 17–18 mmol/L) with low C-peptide (0.172 ng/ml), consistent with type 1 diabetes mellitus; insulin therapy was initiated, but frequent hypoglycemic episodes ensued. At age 17, he developed fatigue, hyponatremia (133–134 mmol/L), low cortisol (48–52 ng/ml), low T3/T4, and mild pituitary enlargement, consistent with hypopituitarism, secondary hypothyroidism, and adrenal insufficiency; prednisone and levothyroxine were prescribed. Between ages 19–20, he experienced hypoglycemic coma, bradycardia, and third-degree atrioventricular block; Electrocardiogram (ECG) and laboratory tests revealed elevated alkaline phosphatase and myoglobin with normal creatine kinase. A temporary pacemaker restored sinus rhythm, and KSS was clinically suspected despite initial negative mitochondrial DNA testing. At age 22–23, worsening cardiac conduction abnormalities accompanied by transient vision loss and episodes of unconsciousness led to permanent pacemaker implantation; mitochondrial DNA analysis of a urine sample detected a deletion spanning chrM: 6656–13830 (Figure 2), confirming KSS. At age 24, he developed severe COVID-19 pneumonia complicated by sepsis and type II respiratory failure; despite mechanical ventilation, his condition deteriorated and he died following the withdrawal of life-sustaining treatment.

Mitochondrial DNA analysis was performed on July 30, 2021 (age 23) using a urine sample (specimen ID: 20C238016) at Beijing Mygeno Medical Laboratory. Genomic DNA was extracted, sheared, and ligated to sequencing adapters. Targeted enrichment of disease-associated mitochondrial regions was achieved using biotinylated probes. The enriched fragments were amplified by PCR and sequenced on the Illumina HiSeq 2000 platform. Sequencing-based copy ratio analysis revealed a region of significantly decreased mitochondrial copy number spanning chrM: 6656–13830 (Figure 2). According to the literature, large-scale deletions within the chrM: 8482–13460 region have been reported to be associated with KSS (17). The identified deletion in this patient’s mitochondrial genome corroborated the clinical diagnosis of KSS.

### 2.2 Diagnosis

The patient initially presented with congenital ptosis at birth, followed by progressive bilateral eyelid elevation impairment during childhood and subsequent multisystem involvement, including type 1 diabetes mellitus, multiple pituitary hormone deficiencies, and progressive cardiac conduction abnormalities. The combination of CPEO, pigmentary retinopathy, and complete atrioventricular block was highly suggestive of KSS. Notably, the clinical course was atypical, as marked endocrine dysfunction and cardiac conduction block preceded the definitive onset of retinopathy and external ophthalmoplegia, contributing to a delayed diagnosis. Initial mitochondrial DNA testing using peripheral blood failed to detect large-scale deletions, highlighting the limitations of blood-based samples in identifying mosaic mitochondrial deletions. Subsequent targeted mitochondrial DNA analysis of a urine sample identified a suspected deletion spanning chrM: 6656–13830, overlapping with the chrM: 8482–13460 region commonly associated with KSS as reported in the literature. In conjunction with the characteristic clinical triad and exclusion of alternative neuromuscular and pituitary disorders, a diagnosis of KSS was established despite incomplete molecular confirmation.

### 2.3 Interventions

The patient underwent multidisciplinary management targeting endocrine insufficiencies and cardiac conduction abnormalities. Endocrine treatment consisted of multiple hormone replacement therapies: insulin for type 1 diabetes mellitus (despite marked glycemic variability and recurrent hypoglycemic episodes), oral prednisone for secondary adrenal insufficiency, and levothyroxine for secondary hypothyroidism. Due to heightened insulin sensitivity and fluctuating glucose levels, intensive glucose monitoring and flexible insulin dosing were required, with frequent adjustments during infections and periods of physiological stress. Cardiac complications were initially managed with temporary pacing during episodes of acute heart block, followed by permanent pacemaker implantation in response to progressive conduction deterioration. Supportive mitochondrial-directed therapies, including coenzyme Q10 supplementation and nutritional optimization, were administered, although evidence supporting their disease-modifying effects remains limited. Throughout the course of treatment, alternative diagnoses–such as myasthenia gravis, primary pituitary pathology, and inherited cardiomyopathies–were systematically excluded through serological testing, electrophysiological studies, and imaging evaluations. In accordance with recent clinical guidelines, early pacemaker implantation is considered essential in KSS patients with conduction abnormalities to prevent sudden cardiac death (18). In retrospect, earlier permanent pacing might have reduced the risk during prior syncopal episodes.

### 2.4 Follow-up and outcomes

Over several years, the patient required continuous monitoring of cardiac rhythm, endocrine function, and metabolic status. Despite appropriate hormone replacement and pacemaker therapy, he remained highly vulnerable to metabolic decompensation during infectious episodes. At age 24, he developed severe COVID-19 pneumonia complicated by sepsis and acute respiratory failure. Although initial management with mechanical ventilation and broad-spectrum antibiotics was promptly initiated, his condition rapidly deteriorated–likely due to underlying mitochondrial dysfunction, which impaired both immune response and metabolic resilience. As his clinical status worsened, he was transferred to the infectious diseases department for specialized management, including antiviral therapy. Unfortunately, despite aggressive treatment, he died shortly after the withdrawal of life-sustaining support, in accordance with the family’s wishes.

## 3 Discussion and conclusion

### 3.1 Literature review

**FIGURE 3:**
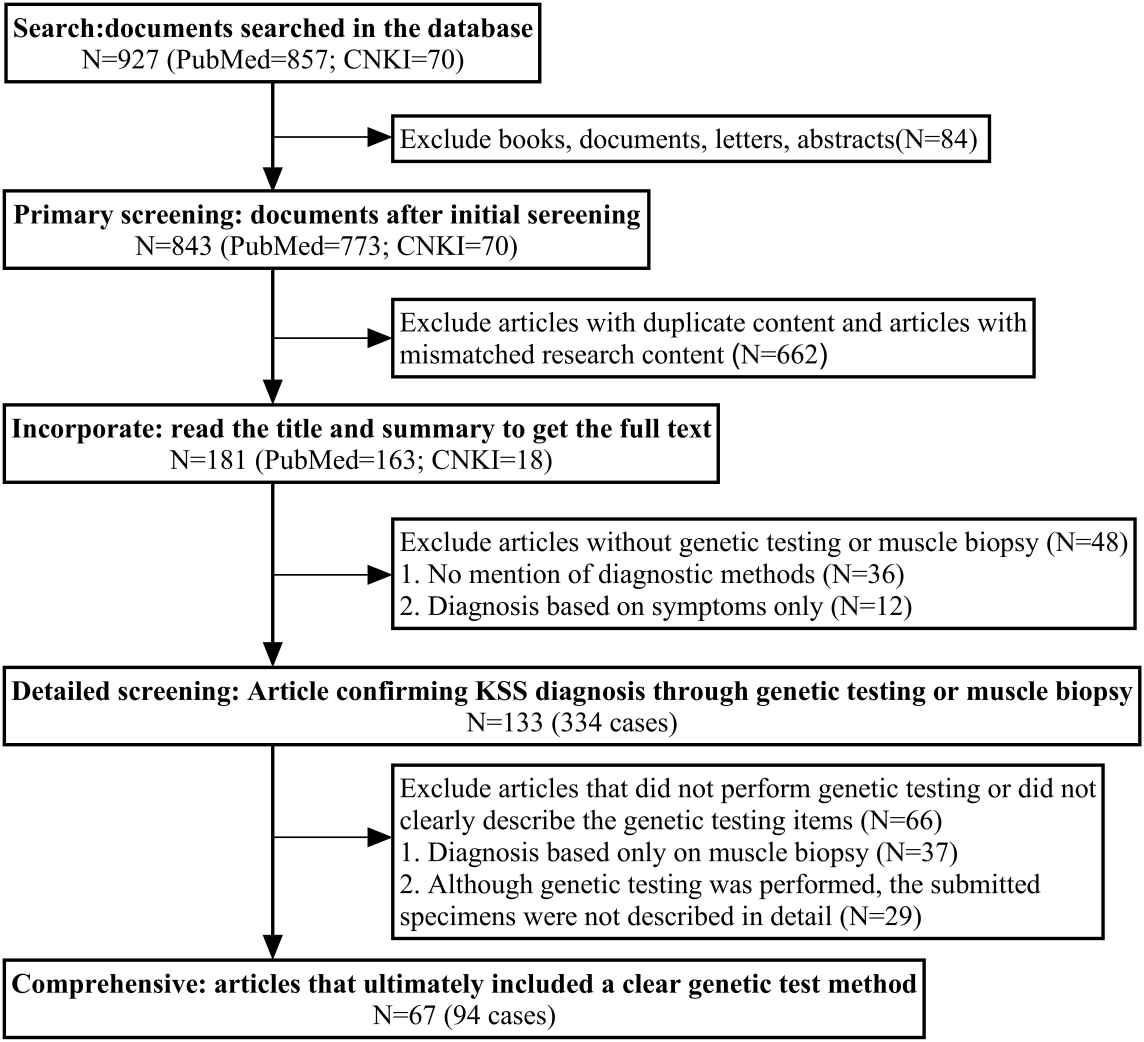
The search flowchart of related article with KSS case reports. China National Knowledge Infrastructure (CNKI); A total of 927 records were retrieved from PubMed and CNKI. After removing duplicates, irrelevant content, and studies without genetic testing or muscle biopsy, 133 articles (334 cases) confirming KSS diagnosis were identified. Among them, 67 articles (94 cases) clearly described genetic testing methods and were included in the final analysis.

We reviewed KSS case reports published in English on PubMed and in Chinese on the China National Knowledge Infrastructure (CNKI) database from 1994 to 2024 (Figure 3), and a total of 133 articles (334 cases) were included, all cases had confirmed diagnoses of KSS and provided information regarding genetic diagnostic approaches. Among these, 67 articles (94 cases) reported the use of genetic testing, with specimens obtained from muscle (*n* = 66), blood (*n* = 42), and urine (*n* = 7). Additionally, 37 articles (55 cases) relied solely on muscle biopsy and microscopic examination for diagnosis, without the use of genetic analysis.

### 3.2 KSS and endocrine function

Endocrine involvement is reported in approximately 35%–67% of patients with KSS (19), typically manifesting as various hormone deficiencies (20). Reported endocrine abnormalities include diabetes mellitus (21), hypopituitarism (22, 23), and short stature (22, 24). Among these, short stature and sexual dysfunction are the most common, with incidence rates of 38% and 20%, respectively (21). Autoimmune thyroiditis and subclinical hypothyroidism are the most frequently observed thyroid disorders in KSS (20).

The prevalence of diabetes mellitus in KSS ranges from 11% to 25% (2, 21). Its pathogenesis is considered multifactorial and is likely attributable to impaired oxidative phosphorylation due to mitochondrial DNA rearrangements, which affect pancreatic islet cell development and function (25). Among diabetic patients, the m.3243A > G mutation is the most frequently identified genetic abnormality (26). Some studies have suggested a potential autoimmune component when KSS was accompanied by multiple endocrine disorders (25). Thyroid dysfunction in KSS is thought to result from mitochondrial damage, leading to impaired oxidative phosphorylation within the thyroid gland and subsequent reduction in thyroid hormone production (21). In this case, the patient initially developed type 1 diabetes, followed by central hypothyroidism and central adrenal cortical insufficiency. The absence of thyroid peroxidase antibodies supports the hypothesis that these endocrine abnormalities were more likely attributable to large-scale mitochondrial DNA deletions.

#### 3.3 KSS and cardiac involvement

Cardiac involvement is the most common manifestation of KSS, affecting approximately 57% of patients and serving as the primary determinant of prognosis. Complete atrioventricular block is among the most severe cardiac abnormalities associated with KSS, with a mortality rate of about 20% (27). Syncope occurs in 45% of KSS patients after cardiac involvement (28), and although syncope can result from multisystem dysfunction (29), cardiac conduction defects remain the primary cause (28). A retrospective case series found that syncope and dilated cardiomyopathy were the most frequent cardiovascular manifestations, observed in 17% of cases (6/35), among whom, 11% (4/35) of the patients died from sudden cardiac death (30). ECG plays a critical role in assessing arrhythmia risk and predicting sudden cardiac death in KSS. Left anterior fascicular block precedes right bundle branch block and may progress to bi-fascicular block, which serves as a marker of impending conduction system failure. The appearance of bi-fascicular block may indicate the need for pacemaker implantation (18). In this case, the patient was diagnosed with left anterior fascicular block at age 12, but his follow-up was irregular. By age 18, the patient developed severe atrioventricular block and experienced recurrent syncope, highlighting the importance of regular ECG monitoring. Current recommendations advocate for annual ECG and echocardiography for KSS patients. Furthermore, criteria for pacemaker implantation should be expanded to prevent sudden cardiac death (31). However, pacemaker use remains underutilized among KSS patients with conduction abnormalities (32). In this case, the patient was admitted following hypoglycemic coma. Although hypoglycemia was rapidly corrected, he remained unconscious. Subsequent ECG revealed third-degree atrioventricular block as the cause of the coma. This highlights the risk of misattributing altered consciousness to hypoglycemia-induced cerebral dysfunction, overlooking potential cardiac conduction abnormalities. We recommend early and continuous cardiac monitoring in KSS patients, with regular ECG evaluations. Pacemaker implantation criteria might be broadened to reduce the risk of sudden cardiac death.

### 3.4 KSS and ocular symptoms

As a mitochondrial myopathy, KSS frequently presents with ocular symptoms, which often appear as early clinical manifestations. The majority of patients develop characteristic ocular features–such as bilateral ptosis, CPEO, and pigmentary retinopathy–typically before the age of 20 (30). Ocular involvement in KSS is heterogeneous and may include ptosis, diplopia, and blurred vision (1), frequently leading to misdiagnosis as other neuromuscular disorders, such as myasthenia gravis (33). In the present case, the patient exhibited ptosis since birth, and fundoscopic examination revealed focal retinal hypopigmentation and discontinuity of the retinal layers at the posterior pole of the right eye. These findings underscore the importance of considering mitochondrial myopathy in the differential diagnosis when patients present with endocrine dysfunction in combination with ocular symptoms.

### 3.5 KSS diagnosis and mitochondrial DNA testing

Genetic testing is essential for diagnosing KSS (34, 35). In this case, peripheral blood mitochondrial DNA analysis failed to detect any abnormalities, whereas urine-based testing identified a large-scale mitochondrial DNA deletion. Based on our literature review (Figure 3), muscle biopsy was the most frequently employed diagnostic method, followed by genetic testing using blood samples, with genetic testing using urine samples being the least applied. Muscle biopsy remains important in evaluating gene variants of uncertain significance. As a post-mitotic tissue, skeletal muscle is closely correlated with clinical phenotypes, making it the preferred tissue for mitochondrial DNA analysis (36, 37). The primary pathological finding in the muscle with KSS is the presence of ragged-red fibers on Gomori trichrome staining (1, 38, 39). However, similar changes may also be observed in aging muscle (20) or in other mitochondrial myopathies (38, 40). A literature review found that 0.024% (3/121) of patients with typical KSS symptoms who underwent muscle biopsy were not definitively diagnosed with KSS. Thus, indicating that muscle biopsy is not always conclusive. Additionally, muscle biopsy is invasive and costly, which further restricts its clinical application. Blood samples, while easily accessible and providing high diagnostic value, are less likely to yield conclusive results in adults over 20 due to the selective depletion of mitochondrial DNA rearrangements in mitotically active cells such as lymphocytes (36, 41, 42). Moreover, the inherent heterogeneity of KSS (14) increases the likelihood of false-negative results in blood-based genetic testing (43). Urine samples exhibit higher and more stable levels of mitochondrial DNA mutations than blood, making them more suitable for genetic testing (41). Varhaug et al. (44) showed that urine samples can replace muscle biopsy for screening KSS patients with single mitochondrial DNA deletions. Muscle biopsy should be reserved for patients with high clinical suspicion of KSS when urine-based testing fails to detect deletions. In the present case, blood genetic testing did not reveal abnormalities, whereas urine analysis confirmed the diagnosis. Based on these findings, we recommend urine-based mitochondrial DNA testing as the preferred initial diagnostic approach for diagnosing KSS, and clinicians should be more attuned to its diagnostic potential in KSS patients.

### 3.6 KSS and hyperlactatemia

This patient also developed hyperlactatemia at age 19. Previous studies have shown that hyperlactatemia is common in KSS patients (45), likely resulting from mitochondrial DNA deletions that impair mitochondrial oxidative phosphorylation (39). Measurement of lactate levels, either at rest or following exercise, may aid with the diagnosis of mitochondrial myopathies (43). Additionally, mitochondrial encephalomyopathy with lactic acidosis and stroke-like episodes (MELAS) syndrome, which often presents with hyperlactatemia (46), can be mistaken for KSS (47). However, MELAS typically involves specific genetic mutations, such as m.3243A > G, and predominantly affects the brain and muscles (46). Although the patient presented with hyperlactatemia, the absence of stroke-like episodes, seizures, or cortical atrophy on neuroimaging, along with negative testing for the m.3243A > G mutation, supported the exclusion of MELAS. Furthermore, the clinical triad of CPEO, ptosis, and third-degree atrioventricular block supported the diagnosis of KSS.

### 3.7 KSS and COVID-19

Patients with KSS, due to mitochondrial defects leading to myopathic damage, may be particularly susceptible to long-term adverse effects on the myocardium, respiratory muscles, and neuromuscular function. Acute stressors, such as infections, can lead to poor clinical outcomes. In this case, the patient developed diabetic ketoacidosis, severe pneumonia, sepsis, and type II respiratory failure following COVID-19 infection, ultimately leading to death, with no similar reports published to date. Moreover, studies indicate that COVID-19 vaccination may induce dysfunction in multiple endocrine glands, including the thyroid, pancreas, pituitary, and adrenal glands (48), potentially exacerbating or inducing pre-existing endocrine disturbances in KSS patients. However, in this case, the patient had not received any COVID-19 vaccinations prior to infection, suggesting that his endocrine deterioration was unlikely to be vaccine-related. Unfortunately, the specific variant of COVID-19 that the patient was infected with could not be determined, as variant testing was not performed. However, based on the timing of the infection, it is likely that the patient was infected with a common variant, such as Delta or Alpha. According to Zhang et al. (49), the fatality rate for the Delta variant was approximately 2.1%, with higher rates observed in patients with underlying conditions, such as Kearns-Sayre syndrome. Given the continued global circulation of COVID-19, clinicians should remain highly vigilant when KSS patients are co-infected with the virus, closely monitoring their multi-system conditions and working to optimize prognosis.

### 3.8 Family perspective

The patient’s family faced significant challenges throughout his treatment journey, including making difficult decisions about life-sustaining interventions and managing the emotional strain of repeated hospitalizations. The decision not to perform an autopsy was particularly difficult, influenced by cultural and personal considerations. Despite these challenges, the family remained actively engaged in the patient’s care and expressed appreciation for the medical interventions, such as pacemaker implantation and hormone replacements, which improved his quality of life during the early stages of the disease.

### 3.9 Limitations

Several limitations should be noted. First, the specific COVID-19 variant infecting the patient could not be identified due to the absence of the test results. According to the time of infection, Delta or Alpha variants were likely but unconfirmed. Second, autopsy was not performed due to family’s refusal, therefore, pathological results of his endocrine and myocardial system were not available. Third, hormone levels during the terminal stage of illness were not quantified.

## Data Availability

The original contributions presented in this study are included in this article/supplementary material, further inquiries can be directed to the corresponding authors.
